# OUTCOMES OF PEOPLE LIVING WITH HIV IN TWO PUBLIC-SECTOR PSYCHIATRIC FACILITIES IN THE EASTERN CAPE USING EFAVIRENZ-CONTAINING REGIMENS

**DOI:** 10.21010/ajid.v14i2.2

**Published:** 2020-07-31

**Authors:** Ilse Truter, Razia Gaida, Christoffel Grobler

**Affiliations:** * Drug Utilization Research Unit (DURU), Department of Pharmacy, Faculty of Health Sciences, Nelson Mandela University, Port Elizabeth, Eastern Cape, South Africa; 1 Department of Psychology, Faculty of Health Sciences, Nelson Mandela University, Port Elizabeth, Eastern Cape, South Africa; † Clinical Manager, Elizabeth Donkin Psychiatric Hospital, Eastern Cape Department of Health, Port Elizabeth, Eastern Cape, South Africa

**Keywords:** HIV-positive, efavirenz-containing regimen, neuropsychiatric, psychiatric, outcomes

## Abstract

**Background:**

People with mental disorders are more vulnerable to human immunodeficiency virus (HIV) infection. A part of first-line treatment for HIV, efavirenz, is routinely avoided in patients with mental illness due to the risk of potential aggravation of the mental illness. This study aimed to determine the outcomes of people living with HIV/AIDS who were mental healthcare users admitted to two public-sector psychiatric facilities and who were prescribed an efavirenz-containing regimen.

**Materials and Methods:**

A retrospective drug utilization study was conducted at two public-sector psychiatric hospitals in the Eastern Cape of South Africa. Patients aged 18 years or older living with HIV were included in the study. Follow-up reviews were conducted at four, 12 and 24 weeks. The patients were seen by doctors at the hospital and notes on progress and medication were recorded in medical records. The files were then reviewed.

**Results:**

A total of 37 patients were enrolled. However, just 26 were reviewed at 24 weeks. A total of 43.2% (n=16) were female patients and the average age of the patients was 39.38±8.76 years. At the baseline, 32.4% (n=12) patients were diagnosed with schizophrenia. A total of 81.08% (n=30) of patients experienced an improvement in psychiatric symptoms after 24 weeks. Of these, 43.3% (n=13) were on an efavirenz-containing regimen.

**Conclusion:**

Majority of the patients demonstrated an improvement in their psychiatric conditions to the extent that they were discharged into the community. This finding suggests that patients with psychiatric disorders on efavirenz can experience stabilisation of their psychiatric symptoms.

## Introduction

People who are mental healthcare users are more vulnerable to human immunodeficiency virus (HIV) infection due to increased risk behaviour, substance abuse and dysfunctional sexual relationships (Johnson *et al.*, 2013). Alternatively, mental disorders may arise as a direct result of HIV infection due to invasion of the central nervous system (CNS) by the virus, psychological distress associated with the infection, or side-effects due to antiretroviral therapy (ART) (Jonhson *et al.*, 2013).

Studies have shown that amongst people living with HIV, depression is the most commonly-reported psychological disorder in South Africa (Freeman *et al.*, 2008; Myer *et al.*, 2008). A study in the South African Province of KwaZulu-Natal (Collins *et al.*, 2009) examined the HIV sero-prevalence of patients admitted to a psychiatric hospital and showed that 26.5% of 151 patients tested were HIV-positive. Furthermore, interviews for the Diagnostic and Statistical Manual (DSM) of Mental Disorders conducted amongst 485 patients at five HIV testing centres in South Africa found that 14.2% of patients were suffering from major depression, 5.0% from generalised anxiety disorder and 19.8% from alcohol abuse (Kagee *et al.*, 2017).

Studies have indicated that people living with HIV (PLWH) who are mental healthcare users have a slower rate of virological suppression than the general population (Chander *et al.*, 2009; Pence *et al.*, 2007; Yehia *et al.*, 2015); suggesting that ensuring adherence to antiretroviral therapy (ART) is more important in this population. A study conducted in Cape Town (Joska *et al.*, 2014) showed a low rate of attendance at follow-up visits following discharge from a psychiatric hospital. Patients who were admitted to the facilities more than once were less likely to attend follow-up visits. However, potential socio-economic factors which may influence the ability of the patient to attend the follow-up visits cannot be ignored (Joska *et al.*, 2014).

The above studies indicate that mental disorders are common amongst PLWH, with the most prevalent being major depressive disorder. This issue calls for integration of mental healthcare services into general wellness programmes surrounding HIV.

Illicit drugs target a wide range of systems within the body, the immune system being one of those affected (Burdo *et al.*, 2006; Cabral *et al.*, 2006). Although the neuropsychological and neuropathological aspects of HIV are understood, those of drugs of abuse are not (Bell *et al.*, 2006). Populations who use illicit substances are at higher risk of being infected with HIV due to needle sharing and unsafe sexual practices (Cabral *et al.*, 2006). Not only do illicit substances pose a hazard in terms of interactions with ART (Antoniou and Lin-in Tseng, 2002; Wynn *et al.*, 2005), there is also a higher concern of non-adherence to ART amongst substance abusers (Chander *et al.*, 2006).

There are a variety of risk factors such as neurotoxicity from the HIV itself and cellular products, the effects of ART on the nervous system, substance abuse, previous neurological conditions, age, the psychological consequences of living with HIV and HIV itself that play a role in the psychiatric status of PLWH (Badkoobehi *et al.*, 2006; Hill and Lee, 2013). Given that neuropsychiatric disorders form part of the clinical picture of HIV, introduction of ART, which has increased the life expectancy of PLWH, has made it more likely that neuropsychiatric conditions will manifest (Dube *et al.*, 2005). The most commonly-diagnosed neurological conditions are minor cognitive disorders, motor disorders and associated HIV-dementia, while the most commonly-reported psychiatric complications are depressive disorders (Dube *et al.*, 2005).

It is well established that efavirenz is associated with inducing neuropsychiatric side-effects soon after its initiation (Gutierrez-Valencia *et al.*, 2011; Kenedi and Goforth 2011; Lochet *et al.*, 2003). Commonly-reported symptoms include: dizziness, insomnia, headache, abnormal dreams and impaired concentration (Arendt, 2006; Kenedi and Goforth 2011; Nelson *et al.*, 2011) which are similar to those of various psychiatric conditions. Clinicians are hesitant to prescribe efavirenz to PLWH who are mental healthcare users largely due to fear of worsening the existing psychiatric condition. In South Africa, the National Consolidated Guidelines for the Prevention of Mother-to-Child Transmission of HIV (PMTCT) and the Management of HIV in Children, Adolescents and Adults (2014) state that efavirenz is contra-indicated in PLWH who are mental healthcare users, and nevirapine or lopinavir/ritonavir should be used instead. In contrast, the package insert of efavirenz does not contraindicate its use in PLWH who are mental health users, but instead, advises caution and to discontinue treatment if severe symptoms manifest.

Efavirenz has been shown to be virologically superior to both lopinavir or ritonavir (Riddler *et al.*, 2008) and nevirapine (Nachega *et al.*, 2008). Due to inhibition of cytochrome P_450_ (CYP_450_) enzyme activity, lopinavir or ritonavir has the potential to increase the serum concentrations of a variety of drugs, particularly those metabolised by the CYP_3A4_ and CYP_2D6_ enzymes of the CYP_450_ system (Cvetkovic and Goa 2003). These include agents such as amitriptyline, buspirone, carbamazepine, paroxetine and risperidone, all of which are used in the treatment of psychiatric conditions, thus requiring dosage adjustments if co-administered (Ogu and Maxa 2000). Nevirapine carries the risk of hepatotoxicity and severe skin reactions (Schouten *et al.*, 2010). Indeed, although its use may be controversial, efavirenz could be safely used in PLWH who are mental healthcare users. It may even theoretically be the preferred choice, given its more favourable side-effect and drug interaction profiles as compared to its alternatives, and would allow for a lower pill burden due to its incorporation into the commonly-used, fixed-dose combinations (Jonhson *et al.*, 2013).

The present study aimed to determine the outcomes of PLWH who are mental healthcare users admitted to two public-sector psychiatric facilities and using an efavirenz-containing regimen.

## Materials and Methods

### Design and setting

A retrospective drug utilization study was conducted at two public-sector psychiatric hospitals in the Eastern Cape Province of South Africa.

### Study population

The medical records of patients were reviewed to obtain the required information. PLWH who were 18 years or older were included in the study. Patients on an efavirenz-containing and efavirenz-free regimens were included, allowing for comparisons to be drawn. Patients were enrolled between July 2014 and February 2015, and were selected according to the inclusion criteria.

### Data collection and analysis

A self-developed data collection tool was piloted and changes made before the baseline information was gathered. The baseline information included gender, age, weight, duration of HIV infection, primary psychiatric diagnosis, psychiatric medication, ART, substance abuse and medical history. Subsequent to the recording of baseline information, follow-up reviews were conducted at four, 12 and 24 weeks. At each of these points, the patient’s progress from the previous follow-up until the current point was evaluated. Patients were seen by the doctors at the hospital and notes on their progress and medication were recorded in the medical record. The records were then reviewed by the researchers using the data collection tool. The information collected included changes in medication, side-effects recorded as well as psychiatric symptom progression. General descriptive statistics were calculated and, considering the small sample sizes, Fisher’s exact test was applied.

### Ethical Considerations

The study was granted ethics clearance by the Nelson Mandela University (H14-HEA-PHA-001) as well as the Eastern Cape Department of Health. The necessary permissions were obtained from the management of each participating hospital before the commencement of data collection. As patients were not directly involved in the study, informed consent was not required.

## Results

### Baseline

A total of 37 patients were included in the study. There were 16 (43.2%) female patients and the average age of the population was 39.38±8.76 years with the majority of patients (n=24; 64.9%) being between the ages of 39 and 50 years. The various baseline (upon admission) diagnoses are summarised in Table 1.

**Table 1:** Gender distribution of psychiatric diagnosis at baseline.

Diagnosis	Number of female patients	Number of male patients
Bipolar disorder	3	5
Cognitive disorder	-	1
Dementia	1	2
Mild mental retardation	1	-
Psychosis secondary to a general medical condition (HIV)	9	3
Schizophrenia	2	10
**Total**	16	21

The various ART regimens prescribed to the study population are outlined in Table 2. Upon admission, there were 17 patients (45.9%) using an efavirenz-containing regimen and 20 patients who were not. These patients were instead using nevirapine (n=13) or a protease inhibitor (n=6), while one patient was not on ART at the time of the study. A total of 16 patients had no record of how long they had known of their HIV-positive status. 29.7% (n=11) of the patients were diagnosed for six years and longer, whereas the remaining 10 patients were all diagnosed as HIV-positive between one and five years before the current hospital admission.

**Table 2:** Number of patients on the different ART regimens.

Regimen	Number of patients
Fixed dose combination (tenofovir, emtricitabine and efavirenz)	11
Efavirenz, lamivudine and tenofovir	5
Efavirenz, lamivudine and zidovudine	1
Nevirapine, lamivudine and tenofovir	9
Nevirapine, lamivudine and zidovudine	4
Lopinavir/ritonavir, lamivudine and tenofovir	2
Lopinavir/ritonavir, lamivudine and stavudine	1
Lopinavir/ritonavir, lamivudine and zidovudine	3
None	1
**Total**	37

A history of tuberculosis infection was recorded for 11 (29.7%) patients and 22 (59.4%) patients with a self-reported history of substance abuse. The most commonly-abused substances were alcohol (n=14; 63.6%) followed by marijuana (n=12; 54.6%) and nicotine (n=4; 18.1%). A total of four patients suffered from epilepsy, one had a previous head injury, and one patient had a history of meningitis.

In addition to ART, patients were receiving a variety of psychiatric medications, often in combination. The choice of medication may be, at times, restricted due to public-sector facilities making use of a standardised medicines formulary. The various psychiatric medication prescribed is summarised in Table 3. Risperidone and sodium valproate were the two most commonly-prescribed agents.

**Table 3:** Psychiatric medication used in HIV-positive patients.

Drug classes	Number of patients
Antiepileptics	15
Atypical antipsychotics	24
Benzodiazepines	7
Lithium	2
Selective-serotonin reuptake inhibitors	2
Tricyclic antidepressants	1
Typical antipsychotics	12

### Follow-up

Only 26 (70.28%) out of 37 patients were still in hospital for review at 24 weeks. During the follow-up period, two patients had absconded, four had been transferred to another psychiatric facility, one had been down-referred to the local clinic for further management, one patient was granted a leave of absence for six months, two patients had no information available and one patient had committed suicide.

As the patients’ mental status improved, the psychiatric diagnosis was, at times, altered upon subsequent evaluation. Therefore, the final diagnoses are summarised in Table 4.

**Table 4:** Gender distribution of psychiatric diagnosis after follow-up.

Diagnosis	Number of female patients	Number of male patients
Bipolar disorder	2	6
Dementia	-	1
Intellectual disability	1	-
Mild neurocognitive disorder	-	1
Psychosis secondary to HIV	6	3
Schizophrenia	2	4
**Total**	11	15

There were 21 (80.8%) patients who experienced an improvement in psychiatric symptoms after 24 weeks. Of these, nine (42.9%) patients were on an efavirenz-containing regimen. One patient was switched from efavirenz to nevirapine, but no improvement was shown at 24 weeks.

The average hospital stay was 13±10 weeks. The large standard deviation was due to three patients who had longer hospital stays of 20, 28 and 36 weeks, respectively. A total of 11 (42.3%) patients were discharged by the end of 24 weeks. Of these 11 patients, five were discharged still using an efavirenz-containing regimen, while the remaining six patients were all prescribed nevirapine. A comparison between patients using efavirenz-containing regimens and those using other regimens is provided in Table 5 below. Fisher’s exact test showed that the differences were not statistically significant with p-values being above 0.05 for all three categories.

**Table 5:** Comparison between patients using efavirenz-containing regimens and those using other regimens.

Outcome	Patients on efavirenz (n=16)	Patients not on efavirenz (n=21)
Patients remaining at the hospital for 24 weeks (p=0.1449)	43.8%	66.7%
Stabilisation of psychiatric symptoms (p=0.2703)	56.3%	71.4%
Substance abuser (p=0.2535)	68.8%	52.4%

## Discussion

Of the 26 patients reviewed for 24 weeks, 11 (42.9%) experienced stabilisation of psychiatric symptoms whilst on efavirenz and were discharged. One patient committed suicide in hospital. The patient was admitted less than four weeks before the incident and was receiving a combination of tenofovir, lamivudine and nevirapine but, known to be non-adherent. The diagnosis made was psychosis secondary to HIV. It is possible that due to non-adherence to ART, the viral load was not suppressed, and the virus had invaded the CNS, resulting in a psychiatric manifestation. However, the patient could also have been suffering from a pre-existing organic mental disorder which went undetected at the primary healthcare level.

When assessing a patient, the clinician had to take into account various causes of the psychiatric symptoms. It is a challenge to determine whether psychiatric disorders in PLWH are incidental, or if they are brought about by HIV (Badkoobehi *et al.*, 2006). HIV infection itself may be associated with psychotic symptoms through viral invasion of the CNS, with new-onset psychosis occurring in 10 to 15% of PLWH (Owe-Larsson *et al.*, 2009). This tends to happen more frequently in those with late-stage disease and those suffering from HIV-associated dementia which constitutes the diagnosis ‘psychosis secondary to HIV’ often seen in this study. If the psychiatric symptoms are indeed brought about by the HIV itself, given that efavirenz has been shown to penetrate the blood brain barrier and virologically superior to its alternatives, it may be the best therapeutic option for the patient. Such patients must be closely monitored. It is noted that some of the patients included in this study were initiated on ART at primary healthcare facilities. This suggests a need for mental healthcare services to be integrated into the HIV services provided at primary healthcare level in order for healthcare workers to identify potential mental disorders and manage or refer patients appropriately.

Interactions between ART and drugs of abuse are not well understood. The current study showed that alcohol was the most commonly-abused substance. Alcohol is metabolised primarily by alcohol dehydrogenase followed by aldehyde dehydrogenase. The acute ingestion of alcohol results in the inhibition of the enzymes CYP_2D6_ and _2C19_, while the long term use of alcohol can induce the activity of CYP_2E1_ and CYP_3A4_. Therefore, the long-term use of alcohol can result in chronic medications like NNRTIs becoming sub-therapeutic (Antoniou and Lin-in Tseng 2002; Wynn *et al.*, 2005). The use of efavirenz by a patient who is chronic user of alcohol, may lead to decreased plasma levels of efavirenz, resulting in HIV in the CNS to proliferate, thereby increasing the risk of the patient developing HIV-associated neurological disorders (HAND) or HIV-associated dementia (HAD). The primary means of metabolism for marijuana or tetrahydrocannabinol (THC) is CYP_3A4,_ meaning that the use of efavirenz may result in THC toxicity, due to the inhibitory activity of efavirenz on CYP_3A4_ (Antoniou and Lin-in Tseng 2002; Wynn *et al.*, 2005). Given that more than half of the population of the current study were marijuana users, it is possible that an interaction with efavirenz occurred and resulted in psychotic symptoms. Considering that PLWH are abusing various substances, further research into their interactions with ART would be enlightening. Illicit drugs are themselves damaging to the CNS and, when combined with HIV infection, the resulting damage to the CNS can be substantial.

It is notable that the South African Department of Health has made the decision to replace efavirenz with dolutegravir in the fixed-dose combination as first-line treatment. Dolutegravir is preferred due to its higher resistance barrier, better adverse-effect profile and more rapid viral suppression as compared to efavirenz which is now relegated to second-line treatment (World Health Organization 2019). However, in spite of the World Health Organization’s (WHO) approval of dolutegravir as first-line treatment, reports of a risk of birth defects associated with dolutegravir in Botswana resulted in a delay in the rollout while further monitoring is ongoing (Gonzalez 2019). In the instance where dolutegravir cannot be prescribed due to safety concerns, WHO (2019) has recommended the use of efavirenz 400mg instead, indicating that efavirenz still has a place in the treatment of HIV. Until dolutegravir is rolled out and widely utilized in South Africa, the impact of its use cannot be determined.

## Conclusion

The majority of patients studied demonstrated an improvement in their psychiatric conditions to the extent that they were discharged from the facilities. Half of these patients were using an efavirenz-containing regimen which shows that patients with psychiatric disorders on efavirenz can experience stabilisation of the acute psychiatric symptoms. More than half of the patients studied were users of marijuana, which suggests substance abuse challenges amongst PLWH who are mental healthcare users. Patients attending primary healthcare facilities are not being adequately screened for psychiatric disorders, and this indicates a need for mental healthcare services to be integrated into the HIV services provided at primary healthcare level. Improved screening tools are needed to adequately identify patients at risk, as well as further studies investigating the re-admission rate of PLWH who are mental health users prescribed an efavirenz-containing regimen would be of interest. Given that efavirenz is part of the fixed dose combination, it is unlikely that its use will decrease, particularly in resource limited settings such as sub-Saharan Africa. It is, therefore, important to determine the safety of efavirenz in PLWH who are mental healthcare users, both at ART initiation and during treatment. This would serve to reduce pill burden and possibly improve adherence to treatment. Integration of mental healthcare services at primary healthcare facilities will enable holistic care of the patient and possibly prevent future hospitalisation.

**List of abbreviations:**
Antiretroviral therapy: –ART
Central nervous system: – CNS
Cytochrome P450: – CYP450
HIV: -associated neurological disorders-HAND
HIV: -associated dementia –HAD
Human immunodeficiency virus: – HIV
People living with HIV: – PLWH
Tetrahydrocannabinol: – THC
